# ZnCr_2_S_4_: Highly effective photocatalyst converting nitrate into N_2_ without over-reduction under both UV and pure visible light

**DOI:** 10.1038/srep30992

**Published:** 2016-08-03

**Authors:** Mufei Yue, Rong Wang, Nana Cheng, Rihong Cong, Wenliang Gao, Tao Yang

**Affiliations:** 1 College of Chemistry and Chemical Engineering, Chongqing University, Chongqing 400044, People’s Republic of China

## Abstract

We propose several superiorities of applying some particular metal sulfides to the photocatalytic nitrate reduction in aqueous solution, including the high density of photogenerated excitons, high N_2_ selectivity (without over-reduction to ammonia). Indeed, ZnCr_2_S_4_ behaved as a highly efficient photocatalyst, and with the assistance of 1 wt% cocatalysts (RuO_*x*_, Ag, Au, Pd, or Pt), the efficiency was greatly improved. The simultaneous loading of Pt and Pd led to a synergistic effect. It offered the highest nitrate conversion rate of ~45 mg N/h together with the N_2_ selectivity of ~89%. Such a high activity remained steady after 5 cycles. The optimal apparent quantum yield at 380 nm was 15.46%. More importantly, with the assistance of the surface plasma resonance effect of Au, the visible light activity achieved 1.352 mg N/h under full arc Xe-lamp, and 0.452 mg N/h under pure visible light (λ > 400 nm). Comparing to the previous achievements in photocatalytic nitrate removal, our work on ZnCr_2_S_4_ eliminates the over-reduction problem, and possesses an extremely high and steady activity under UV-light, as well as a decent conversion rate under pure visible light.

Heterogeneous photocatalysis over semiconductors allows the utilization of solar energy in various chemical reactions, among which overall water splitting is probably the most popular one owing to the global energy crisis^1^,^2^,^3^,^4^. A great amount of research papers have been published, prompting the fast development along this line. On the other hand, the photocatalytic removal of nitrate ions in aqueous solution is comparatively less investigated. The reasons include that it is very hard to find a suitable photocatalyst fulfilling both high efficiency and high N_2_ selectivity because NO_3_^−^ is soluble and highly stable in aqueous solution, in addition, the rather complicity in mechanism of nitrate reduction^5^,^6^,^7^,^8^,^9^,^10^,^11^,^12^,^13^, compared to water splitting, also contributes to the less achievements in this research field.

The World Health Organization recommended the maximum nitrate (NO_3_^−^) concentration of 10 ppm (calculated by nitrogen weight) in ground water, which is the major source of drinking water. Highly concentrated nitrate is toxic to human health and methods for removal of excess NO_3_^−^ from ground water are therefore desired, serving the purpose of environmental protection. Liquid-phase catalytic nitrate hydrogenation has been extensively studied since 1989, when a Pd-Cu bimetal cocatalyst was found to be active by Tacke and Vorlop^14^. In liquid-phase catalytic systems, there are several major problems unsolved before practical applications, including the formation of undesired ammonia, the safety of using hydrogen (reducing agents), and the less efficient contact among solid-liquid-gas phases^7^,^14^,^15^,^16^,^17^,^18^.

Potentially, heterogeneous photocatalysis was suggested to be a green and low cost operation for reducing NO_3_^−^ pollution in ground water. Ideally, NO_3_^−^ reduction occurs on the surface of photocatalyst particles in successive reactions, from NO_3_^−^ to NO_2_^−^ and then from NO_2_^−^ to N·. Every two so-produced nitrogen radicals will further combine to form N_2_, which then leaves the catalyst surface into the gas phase. However, before its industrial application, several issues need to solved, for example, one is to avoid the over-reduction to NH_3_, as it did occur in many cases.

To date, most efforts were devoted to semiconducting TiO_2_ (modified or unmodified)^5^,^6^,^8^,^9^,^11^,^12^,^19^,^20^,^21^,^22^,^23^,^24^,^25^,^26^,^27^,^28^,^29^,^30^,^31^,^32^,^33^,^34^,^35^,^36^,^37^,^38^,^39^,^40^, together with a few other photocatalysts, including SrTiO_3_^21^, K_4_Nb_6_O_17_^21^, ZnS^41^, KTaO_3_^42^, K_*x*_Ga_*x*_Sn_8−*x*_O_16_ (*x* = 1.8)^43^, BaLa_4_Ti_4_O_15_^13^,^14^, and NaTaO_3_^44^. The first problem is that hitherto studied photocatalysts mostly have wide bandgap energies. Then they could be only active under UV-light excitation, moreover, the relatively negative conduction band (CB) potential allow the catalytic reduction of water, and the so-produced hydrogen would over-reduce the nitrate to ammonia^8^,^10^,^11^,^12^,^13^,^16^,^19^,^20^,^22^,^23^,^24^,^25^,^26^,^27^,^28^,^30^,^32^,^33^,^34^,^35^,^36^,^37^,^38^,^39^,^41^,^42^,^45^,^46^,^47^,^48^. In fact, most of such photocatalysts seems more efficient for water reduction than NO_3_^−^ reduction. The only one exception and the milestone work of Ag/TiO_2_ was conducted by F. X. Zhang and his co-workers, where the conversion rate of nitrate achieved approximately 50 mg N/h with an inner irradiation of UV-light and most importantly, the N_2_ selectivity is close to 100%. However, a recent study shows that the easy-oxidation problem of Ag nanoparticles on the surface lead to the fast degradation of the activity for Ag/TiO_2_ catalyst^39^.

Another question is whether the usage of sacrificial agents or additives for pH adjustment is necessary. During the past thirty years, only a few articles reported photocatalytic nitrate reduction without using any additives^19^,^22^,^42^,^45^, however, the disadvantages are so obvious, including the very low efficiency and low N_2_ selectivity, and over-reduction to NH_3_. So, the researchers in this field incline to increase the photocatalytic efficiency by adding an appropriate amount of sacrificial agent (like formic acid, sodium formate, oxalic acid and methanol, *et al*.), and then to dispose these residual additives by additional post-treatments. This situation is quite different with the case in photocatalytic H_2_ generation, where the overall water splitting is highly desired, and the usage of sacrificial agents is considered to be uneconomic.

The last problem is to develop visible light responsive catalysts. Only a few reports claimed visible light activity for nitrate reduction, and some of them are plausible^34^,^47^,^48^. Until recently, Ag-modified TiO_2_ nanocrystals with co-exposed {001}/{101} facets exhibited observable activity of nitrate reduction under simulated solar illumination^40^. Y. Kamiya and his co-workers has developed a dual-catalyst system, consisting of photocatalytic Pt/SrTiO_3_:Rh and non-photocatalytic SnPd/Al_2_O_3_^10^. The photocatalyst utilized the energy from visible light photons and produce H_2_ from water (using methanol as sacrificial agent), which further acted as the reducing agents to convert NO_3_^−^ to N_2_ on the surface of SnPd/Al_2_O_3_ particles. The problem is that the efficiency is very low, 0.09 mg N/h under visible light irradiation (λ > 420 nm). Apparently, TiO_2_ and SrTiO_3_ are UV-light active photocatalyst, therefore it is more effective to develop an intrinsic visible light photocatalyst to gain high density of photogenerated electrons.

Metal sulfides become naturally good candidates as single-phase catalysts to solve most above-mentioned problems, as far as the photo-corrosion problem can be fixed by adding sacrificial agent. First, they usually provide narrower bandgap energies comparing to metal oxides and this is advantageous to possess a higher density of photogenerated electrons, satisfying the first requirement for reducing NO_3_^−^. Second, some particular metal sulfides with insufficient negative CB position prohibit the generation of hydrogen from H_2_O and thus may prevent the over-reducing of NO_3_^−^ to ammonia (it is even toxic than nitrate ions). In fact, some preliminary attempts have been done with two chalcopyrite type sulfides (CuInS_2_ and CuFe_1−*x*_Cr_*x*_S_2_, 0 ≤ *x* ≤ 0.4)^49^,^50^,^51^. Although CuFe_0.7_Cr_0.3_S_2_ possesses a very narrow bandgap (0.8 eV), its optimal activity is pretty low (0.018 mg N/h) under full arc Xe-lamp irradiation. We deduced that the adsorption-desorption of nitrate or nitrite ions to the catalytic sites is vitally important, which is more complex than that in photocatalytic water splitting. For example, simply using narrow bandgap sulfides (like CdS) would not lead to a satisfied catalytic performance for nitrate removal^46^,^47^.

Here in this report, spinel ZnCr_2_S_4_ prepared by solid-state reaction indeed shows a very high photocatalytic activity of NO_3_^−^ reduction. A systematic investigation on the photocatalytic performance of cocatalyst-decorated ZnCr_2_S_4_ samples was performed to obtain an optimal efficiency. Its differential UV-light activity was as high as 45 mg N/h together with 89% N_2_ selectivity under 125 W Hg lamp irradiation, which is very close to the record of Ag/TiO_2_. The activity of ZnCr_2_S_4_ remained steady after 5 cycles. Moreover, it shows a decent visible light activity, i.e. 1.352 mg N/h under full arc Xe-lamp, and 0.452 mg N/h under pure visible light (λ > 400 nm) irradiation. Under all the photocatalytic conditions throughout our study, we did not observe any production of ammonia nor H_2_. Moreover, the apparent quantum yield (AQY) at 380 nm was recorded as 15.46% (with the irradiation beam density of 0.63 W). Note that it is the first time to study the AQYs in the area of photocatalytic nitrate removal. We developed a good single-phase photocatalyst with pure visible light activity, and probably proved a guidepost to develop a new generation of photocatalysts for NO_3_^−^ removal in ground water.

## Results and Discussion

### Phase identification

ZnCr_2_S_4_ crystallizes in the cubic spinel structure with the lattice parameter *a* ~ 10 Å. In literature, the study on ZnCr_2_S_4_ mostly focused on its complicated but interesting magnetic property, for instance, two subsequent magnetic transitions were observed: the first one to an incommensurate helical antiferromagnetic order at 14 K and the second one to a coexisting commensurate spin order at 7 K^52^,^53^. Hence, to the best of our knowledge, it is the first report about the photocatalytic property of ZnCr_2_S_4_.

**Figure 1:**
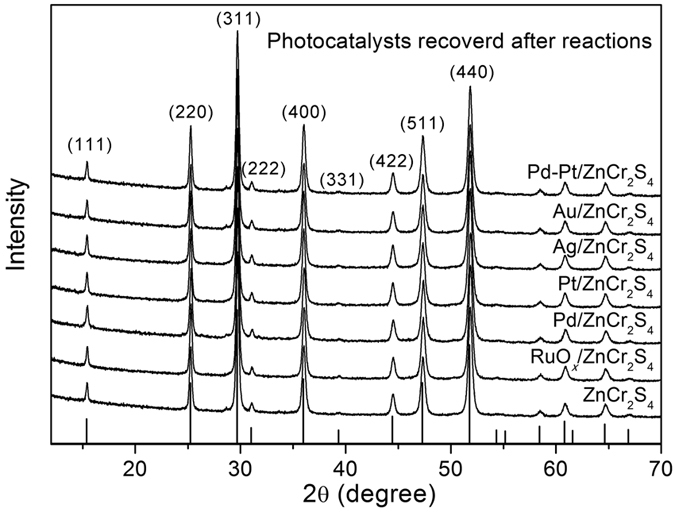
Powder XRD patterns for as-prepared ZnCr_2_S_4_ and the recovered catalysts with loading of cocatalysts. The standard reflections were listed below.

**Figure 2:**
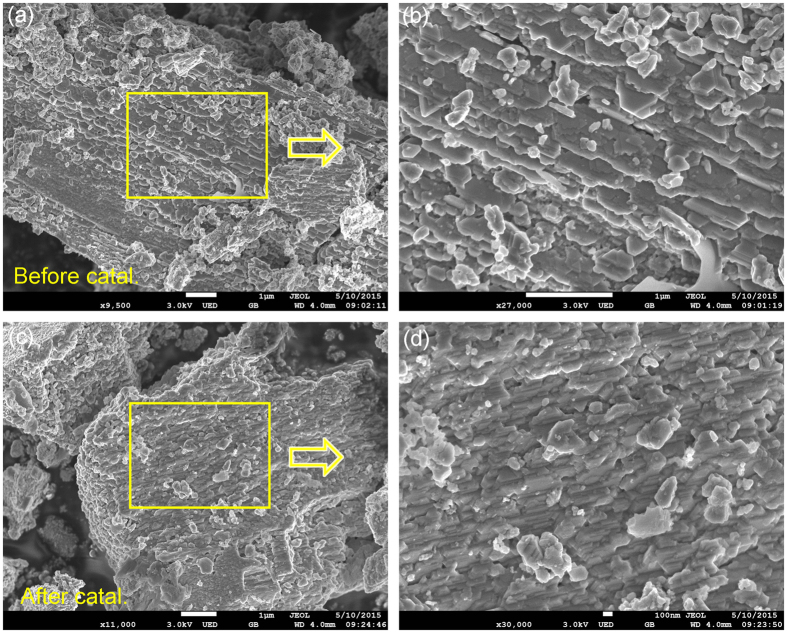
SEM images of ZnCr_2_S_4_ before and after the photocatalytic reactions.

The polycrystalline sample was prepared simply by heating Cr_2_S_3_ and ZnS in an evacuated tube furnace at 700 °C. As indicated by Fig. 1, the phase purity is confirmed by powder XRD. All reflections show a perfect match with the standard pattern. In addition, the recovered photocatalysts showed almost the same patterns to the initial one, which indicates ZnCr_2_S_4_ was very stable during photocatalysis. The relatively broad reflection peaks suggest the possible nano-morphology. Figure 2 presents the SEM images for ZnCr_2_S_4_ catalyst before and after the photocatalytic reactions. The particles are composed of numerous submicron crystallites, most of which possess obvious crystal facets as shown in the enlarged images (Fig. 2b,d). Moreover, the SEM results are consistent with the XRD experiments that all catalysts used in the photocatalytic reactions show neglectable degradation.

UV-Vis DRS spectroscopy was applied to evaluate its bandgap energy. As shown in Supplementary Fig. S1, the deep-brown powder shows a strong light harvesting ability over the visible light region. For most semiconductors, the dependence of the absorption coefficient α on the bandgap energy E_g_ can be expressed by the following equation: αhv = A(hv-E_g_)^n/2^, where h, v, and A are Planck constant, light frequency, and proportionality, respectively; n is determined on the basis of the transition type (i.e. n = 1 for direct transition, n = 4 for indirect transition). Supplementary Fig. S1b provides the plot of (αhv)^2^ against hv (assuming it is a direct transition). The extrapolated value of hv at α = 0 gives an absorption edge energy corresponding to E_g_, which is 1.96 eV for ZnCr_2_S_4_.

### Photocatalytic activity of ZnCr_2_S_4_

**Figure 3:**
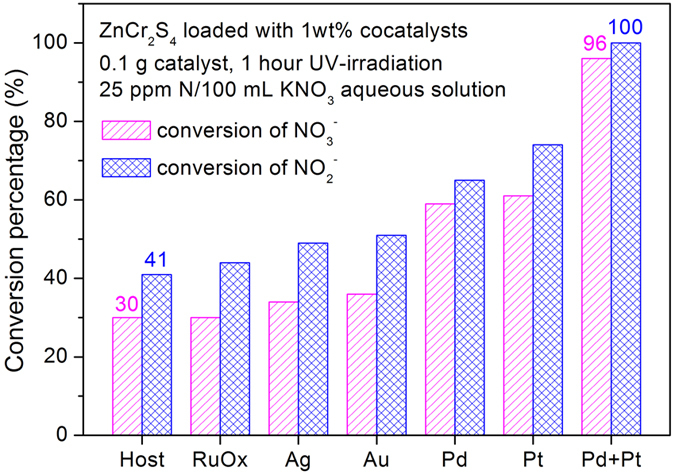
Photocatalytic reduction efficiency for NO_3_^−^ and NO_2_^−^ under UV irradiation for 1 h.

Figure 3 and Supplementary Table S1 shows the respective conversion rates for various catalysts in either 100 mL NO_3_^−^ or NO_2_^−^ aqueous solutions (25 ppm N, calculated by nitrogen weight). The unmodified ZnCr_2_S_4_ shows substantial activities for photocatalyzing the reduction of nitrates and nitrites, *ca*. 0.75 and 1.03 mg N/h, respectively. In addition, the final resultant aqueous solution was checked by inductively coupled plasma-atomic emission spectrometry (ICP-AES) and no chromium was detected, further supporting the high stability of the catalyst.

### Photocatalytic mechanism and activity enhancements by cocatalysts loading

**Figure 4:**
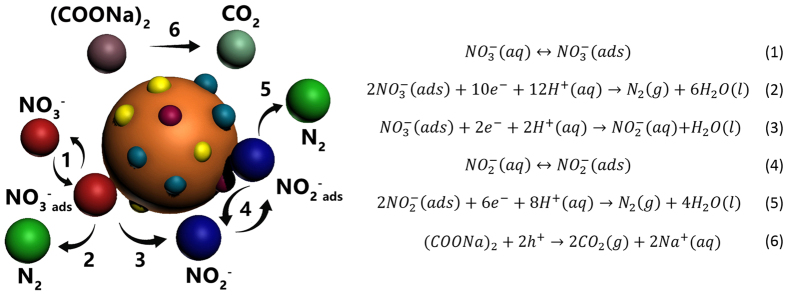
Scheme of photocatalytic nitrate reduction over ZnCr_2_S_4_ loaded with cocatalysts in the presence of the sacrificial agents. The abbreviations of aq, ads, and g in brackets mean ions in the aqueous solution, adsorbed on the surface of the catalyst and in the gas form.

With regards to the photocatalytic mechanism, we propose the reactions occur on the surface of the catalyst particles as shown in Fig. 4. Nitrate ions were first adsorbed to be NO_3_^−^ (ads) as indicated by eq. (1). Then it can be either deeply reduced by 10 e^−^ to N_2_ (g) (see eq. (2)), or just reduced by 2 e^−^ to NO_2_^−^ (aq) and finally leave the surface (see eq. (3)). Apparently, the deep reduction to N_2_ is quite difficult, which needs a significantly high density of electrons on the surface. It is the major reason why most catalysts cannot show high N_2_ differential selectivity in literature. We also need to mention that no ammonia or H_2_ was observed, no matter which cocatalyst was loaded in our study. In the following, the nitrite ions in the aqueous solution could be further adsorbed to be NO_2_^−^ (ads) and reduced by 8 e^−^ to the final product N_2_ (g) (see eqs (4) and (5)). The corresponding h^+^ with positive charge were consumed by oxalic ions (see eq. (6)).

For heterogeneous photocatalysts, it is a common strategy to enhance the efficiency by loading cocatalysts to the particle surface. Appropriately loaded cocatalyst particles can serve as either electron or hole collectors, which could facilitate the spatial separation of photogenerated charges, and therefore allow a higher density of charges at the catalytic sites. Meanwhile, in the reduction reaction of nitrates/nitrites, the adsorption and desorption of substrate ions at catalytic sites is a dynamic equilibrium process. The loaded cocatalyst particles would also increase the number of binding sites and delay desorption of substrate ions by lowering the potential barrier. This could increase the conversion rate of NO_3_^−^ and the selectivity of N_2_ as well. We believe that the synergetic improvements on charge separation and binding ability were supposed to increase the photocatalytic efficiency of ZnCr_2_S_4_ for the reduction of NO_3_^−^ in aqueous solution.

As shown in Fig. 3 and Supplementary Table S1, the use of RuO_*x*_ as cocatalyst did not increase the efficiency of nitrate reduction, and only show a slight improvement to the nitrite reduction. When using Ag, Au, Pd and Pt as cocatalysts, the photocatalytic efficiency was enhanced from 0.85 to 1.53 mg N/h for NO_3_^−^ reduction and from 1.23 to 1.85 mg N/h for NO_2_^−^ reduction, respectively. The efficiency for NO_2_^−^ reduction is higher than NO_3_^−^ reduction for each studied catalyst. When loading cocatalysts, the increasing tendency of NO_3_^−^ and NO_2_^−^ reduction is consistent with each other, which is understandable.

We applied a dual-cocatalyst loading to ZnCr_2_S_4_ with 0.5 wt% Pd and 0.5 wt% Pt. The efficiency was significantly enhanced, showing almost a complete conversion of both NO_3_^−^ and NO_2_^−^ (See Fig. 3). It is quite interesting that the total mass of the used dual-cocatalysts was equal to 1 wt%, while it shows a significant higher activity, comparing to those of either 1 wt% Pd or 1 wt% Pt loaded samples. Accordingly, it suggested a synergetic effect between Pd and Pt cocatalysts. XPS spectrum was collected for this Pt-Pd co-loaded photocatalyst (see Supplementary Fig. S2) and both are suggested to be metal. We speculate the synergetic effect occurs when the loaded metal particles are spatially close to each other. The intermediate product (i.e. NO_2_^−^) could be promptly reduced on the metal particles nearby, rather than through desorption and re-adsorption process.

As stated above, the photocatalytic mechanism here was interpreted as surficial catalytic reactions. To support this assumption, we monitored the photocatalytic efficiency of ZnCr_2_S_4_ loaded with both 1 wt% Pd and 1 wt% Pt, when adding additional ions into the aqueous solution. As shown in Supplementary Fig. S3a, the real tap water from our lab was also applied, in which the nitrate conversion rate slighted decreased from 3.42 to 3.10 mg N/h. With the rational incorporation of F^−^, Cl^−^, Br^−^ and I^−^ (all in the concentration of 0.001 M), the conversion rate and the N_2_ selectivity decreased successively. Such a decline in efficiency is more obvious when using the large anion SO_4_^2−^ and H_2_PO_4_^−^. Furthermore, the photocatalytic nitrate conversion and N_2_ selectivity also decreased when increasing the concentration of NaCl (from 0.001 to 0.02 M) as shown in Supplementary Fig. S3b. All these observations imply the photocatalytic reactions occur on the catalyst surface. Similar mechanism was also proposed by previous researchers^5^,^11^,^29^,^32^.

### Treatments of highly concentrated nitrate aqueous solution

**Figure 5:**
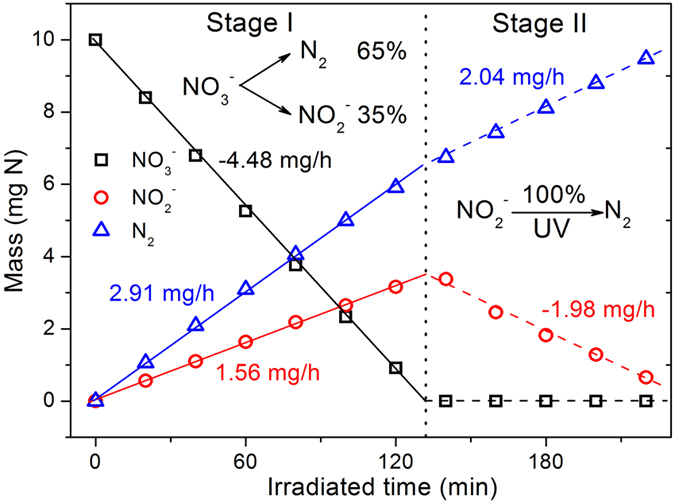
Reduction of 100 mL of an aqueous solution of NO_3_^−^ (100 ppm N) using ZnCr_2_S_4_ loaded with 0.5 wt% Pd and 0.5 wt% Pt. For better understanding and easy comparison, the respective contents of NO_3_^−^, NO_2_^−^ and N_2_ were calculated into the mass of N (mg N). Photocatalytic conditions: 100 mL of NO_3_^−^ aqueous solution containing 100 ppm N, 0.25 g of catalyst, 500 W Hg lamp, in evacuated system.

To understand the photocatalytic process (especially the selectivity of N_2_), we performed an extended reduction experiment in a highly concentrated aqueous solution of NO_3_^−^ (100 ppm N). In this experiment, 0.25 g of ZnCr_2_S_4_ loaded with 0.5 wt% Pd and 0.5 wt% Pt was used. The NO_3_^−^ reduction occurred in two stages (see Fig. 5). In the first stage (roughly the first two hours), only some of the NO_3_^−^ was reduced, and the remaining NO_3_^−^ coexisted with the reduction product NO_2_^−^. In this stage, production of NO_2_^−^ and N_2_ occurred simultaneously. These reactions were apparently zero-order reactions. The observed conversion rate of NO_3_^−^ was 4.48 mg N/h. The production rates of NO_2_^−^ and N_2_ were 1.56 and 2.91 mg N/h, respectively. The calculated differential selectivity of N_2_ was 65%.

When all the NO_3_^−^ was reduced, the second stage began, which only comprises the reduction of NO_2_^−^ to N_2_. The rates for reduction of NO_2_^−^ and production of N_2_ were similar at 1.98 and 2.04 mg N/h, respectively. This reaction was apparently still a zero-order reaction. It should be noted that the N_2_ production rate in the second stage was smaller than that in the first stage. This difference arises because N_2_ in the first stage is produced from the reduction of both NO_3_^−^ and NO_2_^−^, while in the second stage N_2_ is only produced from NO_2_^−^. In other words, in the first stage, some of the NO_3_^−^ species were strongly bound to the catalytic sites and therefore can be deeply reduced to N·. Apparently, the final selectivity of N_2_ could be optimized to 100% simply by extending the irradiation time. The recovered photocatalyst loaded with both Pd and Pt was checked by powder XRD (see Fig. 1) to remain intact after 4 hours UV irradiation.

### Evaluation of apparent quantum yields

In the research field of photocatalytic water splitting, people prefer to use apparent quantum yields (AQYs) to compare the intrinsic activity of a photocatalyst. In fact, AQY is also a semi-quantitative parameter, because people cannot measure the exact number of the photons absorbed by catalyst powder in an aqueous solution. Here we present the AQY study using ZnCr_2_S_4_ in the photocatalytic nitrate reduction.

**Figure 6:**
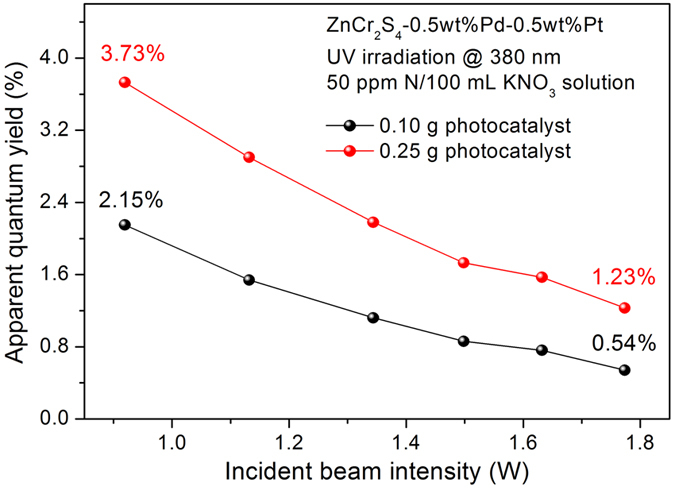
Corresponding apparent quantum yields along with the change of the incident beam intensity for ZnCr_2_S_4_ loaded with 0.5 wt% Pd and 0.5 wt% Pt. The black and red curves represent the AQYs for different quantity of the catalyst used in the reactions.

Under the monochromatic irradiation at 380 nm, the observed AQYs along with the change of the incident beam intensity for ZnCr_2_S_4_ loaded with 0.5 wt% Pd and 0.5 wt% Pt were presented in Fig. 6 and Supplementary Table S2. With the decreasing of the beam light intensity from 1.77 to 0.92 W, the AQY increased from 0.54 to 2.15% (using 0.1 g photocatalyst) or from 1.23 to 3.73% (using 0.25 g photocatalyst). The opposite changing tendency of light intensity and AQY is understandable, because a higher density of the beam light leads to a higher ratio of the scattered photons. Therefore, when decreasing the irradiated photon density, the AQY value would become closer to the absolute quantum yield.

As shown in Supplementary Table S2, with regard to the N_2_ selectivity, a higher beam intensity would lead to a higher N_2_ selectivity when other experimental conditions remained unchanged. This characteristic can be explained as below. The reduction of nitrate contains two successive reactions, from NO_3_^−^ to NO_2_^−^ and then from NO_2_^−^ to N·. This mechanism is quite different with that of photocatalytic water reduction, where only one electron was needed from H^+^ to H. Herein, the nitrate reduction obviously requires a much higher density of photogenerated electrons in the catalytic sites, and apparently its deep conversion to N_2_ was favored when there was a high density of incident beam.

### Achieve the highest activity under UV-light irradiation

As stated above, the combination of Pt and Pd loading would generate a very high activity probably due to the synergetic effect. In this section, we thus increase the loading content up to 3 wt% for each metal to achieve the highest activity under UV-light irradiation as indicated in Supplementary Fig. S4. The differential conversion and N_2_ selectivity both increase accordingly as we expected. From the industrial view of point, the usage of the noble metal should be as low as possible for the economic purpose. In our case, we did not further increase the content of the cocatalysts, which probably would give an even better performance.

**Figure 7:**
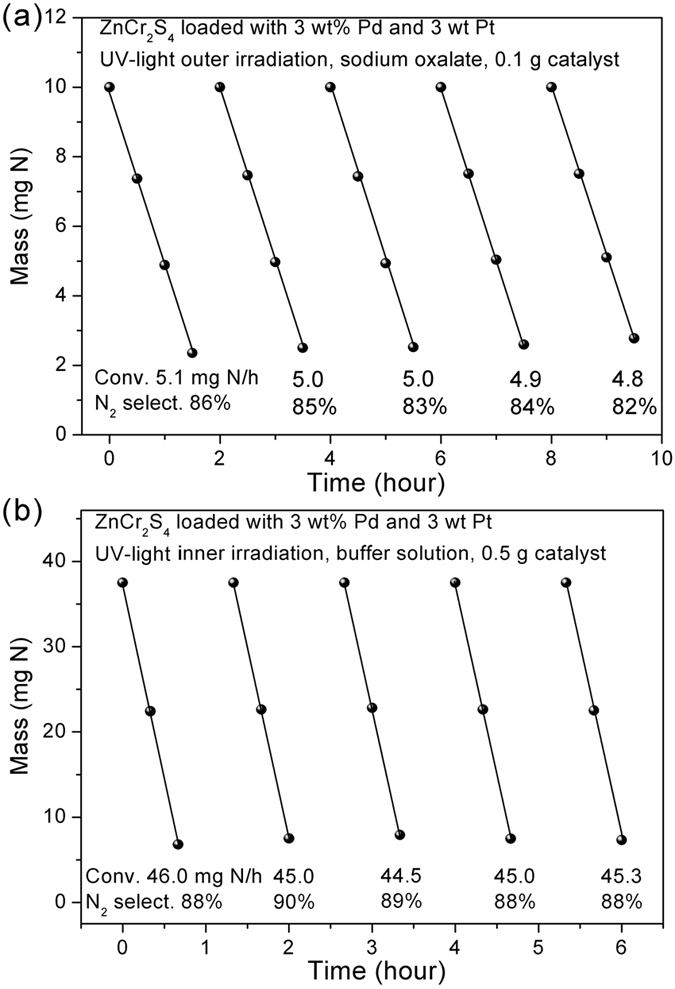
(**a**) Cycling experiments on ZnCr_2_S_4_ loaded 3 wt% Pd- 3 wt% Pt. Photocatalytic conditions: 100 mL of NO_3_^−^ aqueous solution containing 100 ppm N, 0.1 g of catalyst, 500 W Hg lamp, and outer irradiation system. The differential conversion rates and N_2_ selectivity are also presented at the bottom; (**b**) cycling experiments in inner irradiation setup for enhanced activities. Photocatalytic conditions: 200 mL of NO_3_^−^ aqueous solution containing 150 ppm N, 0.5 g of catalyst, 125 W Hg lamp, and in acidic buffer solution.

Here, we need to check the stability of this particular metal sulfide (with 3 wt% Pd and 3 wt% Pt loaded) in both sodium oxalate and acidic buffer solution. We first performed the cycling experiments. After each cycle, the resultant supernatant solution was poured out and the catalyst was washed by water in the reaction vessel twice. New NO_3_^−^ aqueous solution was added into the reaction vessel and after a half-hour dark reaction for adsorption-desorption equilibrium, the UV-light was switched on. As shown in Fig. 7a, after 5 cycles (7.5 hours irradiation in total) the conversion rate slightly decrease from 5.1 to 4.8 mg N/h (about 6% decreasing). 2.7 mmol NO_3_^−^ in total was reduced to either NO_2_^−^ or N_2_, and only 0.34 mmol photocatalyst (ZnCr_2_S_4_) was used. Moreover, the powder XRD pattern after long-term experiments is consistent with the initial one, indicating that it remained as a sulfide. Accordingly, we believe the strategy of using sodium oxalate as the sacrificial agent is effective to prevent the photo-corrosion of ZnCr_2_S_4_ during photocatalytic nitrate reduction. The very slightly decreasing of the activity was attributed to the loss of the catalyst powder during the cycling experiments or the mechanical loss of the loading cocatalysts by stirring.

The highest activity could be achieved by increasing the amount of powder catalyst, using the inner irradiation setup and in acidic buffer solution as shown in Fig. 7b. The activity reaches as high as ~45 mg N/h with the N_2_ selectivity ~89%. This remains steady and is very close to the record of Ag/TiO_2_ (*ca*. ~50 mg N/h) conducted by Zhang and his co-workers (by applying very similar conditions), while the latter show an apparent degradation problem due to the instability of Ag nanoparticles. Moreover, the apparent quantum yields (AQY) at 380 nm for this particular catalyst under the optimal conditions was estimated to be 15.46% (with the irradiation bean density of 0.63 W).

### Visible light activity for nitrate reduction

**Figure 8:**
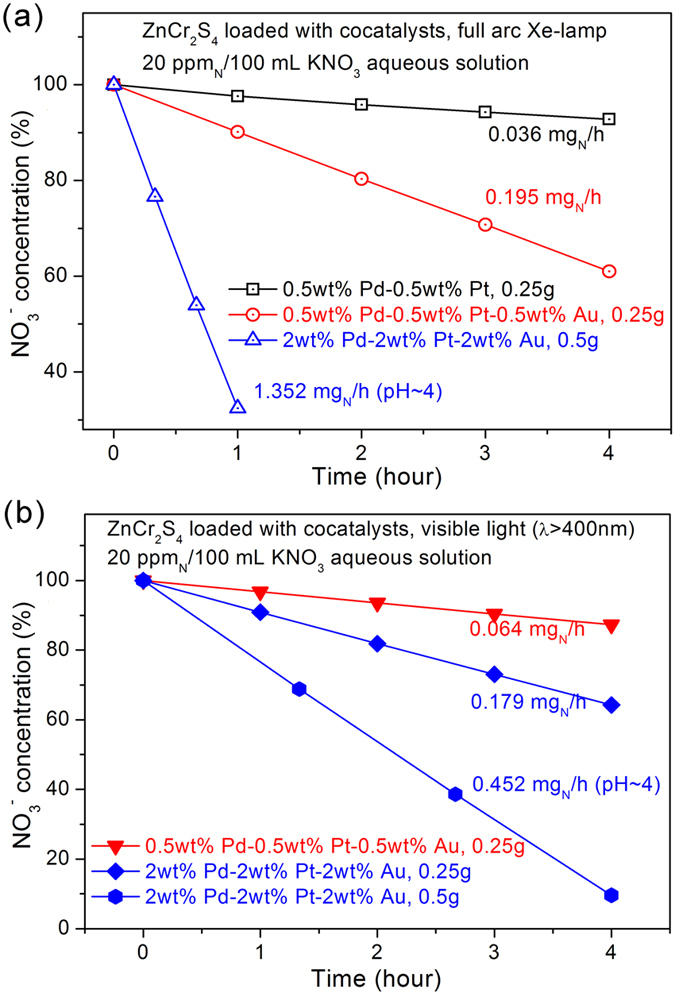
(**a**) Efficiency of the photocatalytic reduction of NO_3_^−^ under irradiation from a full arc Xe lamp. pH ~ 4 means that sodium-formate/formic acid buffer solution was applied; (**b**) the activities under pure visible light by applying a 400-nm cut-off filter to the Xe lamp.

Although the ZnCr_2_S_4_ catalyst exhibited high photocatalytic activities under UV irradiation, the majority of the energy in sunlight comes from visible light. For visible light irradiation, it is advantageous that metal sulfides have a narrow bandgap because of the high potential of the valence band arising from their S 2p orbitals. ZnCr_2_S_4_ could show visible light activity, and we performed a preliminary experiment to investigate this. Photocatalytic reduction of NO_3_^−^ (20 ppm N) was attempted with irradiation from a full arc Xe lamp. The conversion rate was 0.036 mg N/h for ZnCr_2_S_4_ loaded with 0.5 wt% Pd and 0.5 wt% Pt (see Fig. 8a). The photocatalytic activity was significantly lower than that achieved with UV irradiation even though ZnCr_2_S_4_ has a narrow bandgap energy (1.96 eV). This decreasing in the conversion rate could have caused by the low density of electrons when irradiated by the Xe lamp.

It is therefore necessary to enhance the light harvesting ability of our catalyst. In literature, Au nanoparticles can serve as the cocatalyst to improve the photocatalytic performance of TiO_2_ according to the surface plasma resonance (SPR) theory^54^,^55^,^56^, which provide a substantial increase to the absorption of visible light photons. Here in our case, the loading of Au cocatalyst alone on the surface only show a slight increase of the activity compared to the host, when irradiated by UV light (see Fig. 3). As shown in Fig. 8, the catalysts loaded with Pd, Pt and Au (all in the mass fraction of 0.5%) show a much enhanced photocatalytic efficiency (~5 times higher) when irradiated by a full arc Xe-lamp. We believe this great enhancement in activity was due to the SPR effect of Au nanoparticles, which increase the visible light absorption ability of the catalyst, and the catalytic reduction of nitrate ions may still mostly occurs at Pd/Pt sites. The differential conversion rate could further increase up to 1.352 mg N/h, by increasing the amount of the catalyst, the cocatalysts, and using acidic buffer solution as sacrificial agents (see Fig. 8a).

As is known, the full arc Xe lamp may provide some part of UV–light irradiation, thus we have to apply a 400 nm cut-off filter to investigate its pure visible light activity. As shown in Fig. 8b, the conversion rate of nitrate is 0.064 and 0.179 mg N/h for lightly and heavily loaded catalysts, respectively. By increasing the mass of catalyst, the final optimal conversion rate is 0.452 mg N/h in buffer solution (pH ~ 4). Note that the conversion rate of 0.09 mg N/h was recently reported by Y. Kamiya under pure visible light condition, using a dual-catalyst system of Pt/SrTiO_3_:Rh and SnPd/Al_2_O_3_^10^.

### Potential of metal sulfides for photocatalytic nitrate reduction

Comparing to the traditional photocatalyst TiO_2_, metal sulfides (including ZnCr_2_S_4_, CuInS_2_^49^, CuFe_1−*x*_Cr_*x*_S_2_^50^,^51^) all possess an intrinsic narrow bandgap energy, allowing the absorption ability to visible light. The apparent advantage for narrow bandgap is the possible higher density of the charge-excitons, which is important for nitrate reduction reactions as it requires 5 electrons in total from NO_3_^−^ to N·. The selectivity of N_2_ could be improved by technically optimizing the loading of cocatalysts. Efforts are still needed to generate possible heterojunctions to further facilitating the charge separation and light harvesting ability, which usually show superior performances than single-phase catalysts^57^,^58^.

There still remains an unsolved problem. A small amount of sacrificial agents (in the level of tens of ppm) is needed to prevent the hydrolysis or self-oxidation of metal sulfides. In photocatalytic water splitting, this is unacceptable because it is uneconomic comparing to the photovoltaic industry. While for the environmental protection, we first need to reduce nitrate ions, which is very stable in aqueous solution. Thereafter, the residual sacrificial agent, for example, oxalic acid or formic acid, is quite easy to remove, especially in a low concentration. Alternatively, people tend to use other un-harmful molecules to consuming the photogenerated holes, like sucrose or glucose^12^,^25^. Overall, metal sulfides have shown a great success in photocatalytic water reduction, where people devoted all efforts to the band structure and morphology engineering^59^. We believe that the future of metal sulfides in the application of water purification would be also promising.

## Conclusions

In conclusion, we systematically investigated the performance of spinel ZnCr_2_S_4_ in photocatalytic nitrate reduction. The intrinsic narrow bandgap energy (1.96 eV) allow a strong light harvesting ability and indeed the as-prepared ZnCr_2_S_4_ behaved as an efficient catalyst for nitrate reduction under UV light irradiation. Cocatalysts, including RuO_*x*_, Ag, Au, Pd, Pt, were loaded to further improve the reduction efficiency. A synergic effect was observed when loading Pd and Pt, which offered a very high activity. Increasing the loading content would enhance the activity accordingly. The highest nitrate conversion rate of 45 mg N/h together with the N_2_ selectivity of 89% was achieved upon ZnCr_2_S_4_ loaded with 3 wt% Pd and 3 wt% Pt, under the following experimental conditions: inner UV-irradiation (125 W), sodium-formate/formic acid buffer solution. The AQY at 380 nm for this particular catalyst was estimated to be 15.46% (with the irradiation beam density of 0.63 W). Most importantly, the visible light activity was explored for ZnCr_2_S_4_ loaded with three cocatalysts simultaneously, including Pd, Pt and Au nanoparticles. With the assistance of the SPR effect of Au nanoparticles, the optimal conversion rate of nitrate is 1.352 mg N/h under full arc Xe-lamp, and 0.452 mg N/h under pure visible light (λ > 400 nm) irradiation. Our work proved that metal sulfides with appropriate modifications are good candidates for photocatalytic nitrate reduction.

## Methods

### Preparations of the catalysts

Cr_2_S_3_ used in our study is not from commercial source but from a decomposition of a home-made Cr-containing compound. In detail, 2 mmol of CrCl_3_·2H_2_O and 60 mmol of H_2_NCSNH_2_ were mixed with 1.5 g of C_2_H_4_O_4_·2H_2_O evenly and then the mixture was transferred into a 50 mL Teflon in a stainless-steel autoclave and sealed. After reacting at 230 °C for 3 days, a black powder sample was obtained by washing away soluble residuals, and it was further converted into Cr_2_S_3_ by annealing under vacuum at 600 °C for 2 h. Thereafter, a stoichiometric mixture of Cr_2_S_3_ and ZnS (Alfa Aesar, 99.9%) was homogenized using an agate mortar and followed by a heating at 700 °C for 2 h in an evacuated tube furnace. The resultant brown powder was checked to be phase-pure polycrystalline ZnCr_2_S_4_.

The noble metal or metal oxide cocatalyst loading to ZnCr_2_S_4_ was performed using the procedure described below. Typically, 0.20 g of ZnCr_2_S_4_, 1.4 mL of H_2_PtCl_6_·6H_2_O (1.48 mg Pt/mL), and 30 mL of distilled water were placed in a 100-mL beaker. This solution in a 100 mL beaker was processed with an ultrasonic treatment for 20 minutes. An appropriate amount of diluted KBH_4_ aqueous solution was added into the beaker very slowly with constant stirring. Finally, the obtained powder sample was extensively washed by water and dried at 60 °C. For other metal loading, the used sources are RuCl_3_, AgNO_3,_ HAuCl_4_ ·4H_2_O, and PdCl_2_, respectively. It is assumed that most cocatalyst ions in aqueous solution were successfully loaded. The accurate amount of the loading cocatalyst is in fact difficult to determine as the insoluble nature of most noble metal.

### General characterizations

Powder X-ray diffraction (XRD) data were collected on a PANalytical X’pert diffractometer equipped with a PIXcel 1D detector (Cu Kα radiation, 1.5406 Å). The operation voltage and current were 45 kV and 40 mA, respectively. Scanning electron microscopy images were recorded using a JEOL JSM-7800F electron microscope at a working distance of 4.0 mm. UV-Vis diffused reflectance spectra (DRS) were recorded by Shimadzu UV-3600 spectrometer equipped with an integrating sphere attachment. BaSO_4_ was used as reflectance standard. X-ray photoelectron spectra (XPS) were acquired with UK Kratos Axis Ultra spectrometer with Al Kα X-ray source operated at 15 kV and 15 mA. Electron binding energies were calibrated against the C 1 s emission at E_b_ = 284.8 eV to correct the contract potential differences between the sample and the spectrometer.

### Photocatalytic activity evaluation

The photocatalytic activities of the prepared catalysts were mostly tested in a sealed circulation system equipped with a vacuum line (Perfect Light, LabSolar-IIIAG), a 150-ml Pyrex glass reactor, and a gas sampling port that was directly connected to a gas chromatograph (Shanghai Techcomp, GC7900). A 5 °C cycling bath was applied to the reaction vessel to keep the temperature constant and cool. The gas chromatograph was equipped with a thermal conductivity detector and a column packed with 5A molecular sieves. Helium was used as the carrier gas to detect the so-produced N_2_ online. 500 W Hg or 300 W Xe-lamp were used to provide UV- or visible light irradiation (CEL-M500 or CEL-HXF300, Beijing AuLight Ltd. Co.), which was applied from the top of the reaction vessel (See Supplementary Fig. S5).

In most cases, our photocatalytic experiments were performed using the above setup. In some cases, we also applied a setup with an inner irradiation lamp. The photocatalytic reaction was carried out in a double-walled quartz cell cooled by water with a 125 W Hg lamp as the light source. A schematic view of the setup can be found in SI.

KNO_3_ and KNO_2_ were used as the nitrate and nitrite source, respectively. The concentration was calculated by the weight of nitrogen. For example, 100 mL of NO_3_^−^ (NO_2_^−^) aqueous solution containing 50 ppm N was obtained by adding 36.1 mg of KNO_3_ (or 30.4 mg of KNO_2_) into 100 mL of distilled water. Either sodium oxalate (in the concentration of 0.026 mol/L) or an acid buffer solution (the initial concentration of sodium-formate and formic are both 0.035 mol/L) was used as the sacrificial reagent. The respective dosage of sacrificial agent is 2- and 7-times excess (assuming all the carbon in C_2_O_4_^2−^ or COOH^−^ were oxidized to CO_2_). In previous reports, the common usage of sacrificial agent was about 2~15 times excess^5^,^6^,^10^,^40^.

During the reaction, a small amount of the solution was withdrawn periodically, the catalyst was immediately separated by centrifugation, and the upper solution was analyzed to determine the residual concentration of NO_3_^−^ and NO_2_^−^ with an UV-Vis spectrophotometer according to the colorimetric method^60^. Ammonia was not detected throughout our study. For detail, we mixed the Nessler’s reagent (HgCl_2_-KI-KOH aqueous solution) with the sample solution. If there was any ammonia, there should be an absorption centered at 420 nm. The detection limit is 0.015 mmol/L experimentally, and no such absorption was observed in our study and thus, we conclude no ammonia can be produced if ZnCr_2_S_4_ was used as the photocatalyst. In fact, ZnCr_2_S_4_ is unable to photocatalyze the H_2_ generation in any conditions, including using the common sacrificial agent of Na_2_S and Na_2_SO_3_. We believe this might be responsible to the absence of ammonia.

It is known that the photocatalytic activity strongly depends on the applied experimental conditions, including but not limited to the mass of the photocatalyst, the incident beam intensity, the volume and the concentration of the starting aqueous solution, and so on. Please note the photocatalytic efficiency in this work is presented as the reduced amount of nitrate (calculated according to the mass of N) per hour with the unit mg N/h. Unless further stated, the N_2_ selectivity refers to the differential selectivity. In our case, the final N_2_ selectivity could easily achieve 100% by simply extending a few more hours of irradiation.

In real application, the conversion rate and differential selectivity are the common criteria to evaluate the quality of a particular photocatalyst. While, from the fundamental aspect, the apparent quantum yields (AQYs) under a monochromatic irradiation is more meaningful.


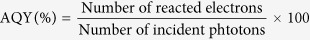


In our study, the number of reacted electrons can be calculated according to the nitrate reduction rate and the N_2_ selectivity, and the number of incident photons can be measured by the Si-photodiode.

## Additional Information

**How to cite this article**: Yue, M. *et al*. ZnCr_2_S_4_: Highly effective photocatalyst converting nitrate into N_2_ without over-reduction under both UV and pure visible light. *Sci. Rep*. **6**, 30992; doi: 10.1038/srep30992 (2016).

## Supplementary Material

Supplementary Information
