# Supplementary material for: ZnCr2S4: Highly effective photocatalyst converting nitrate into N2 without over-reduction under both UV and pure visible light
Source: Sci Rep. 2016 Aug 3;6:30992. doi: 10.1038/srep30992 (PMC4971535; doi:10.1038/srep30992)
Supplement: Supplementary Information [file srep30992-s1.pdf]

Supplementary Information for

$ZnCr_2S_4$: Highly effective photocatalyst converting nitrate into N_2 without over-reduction under both UV and pure visible light

Mufei Yue, Rong Wang, Nana Cheng, Rihong Cong, Wenliang Gao, Tao Yang[*]

College of Chemistry and Chemical Engineering, Chongqing University, Chongqing 400044, People's Republic of China

[*]E-mails: taoyang@cqu.edu.cn.

Table S1 Photocatalytic efficiency for various catalysts during the photocatalytic reduction of NO_3^- and NO_2^- under UV irradiation for 1 h. Experimental conditions: 100 mL of NO_3^-/NO_2^- aqueous solution containing 25 ppm N, 0.1 g of catalyst, 500 W Hg lamp, in sodium oxalate solution.

Co-catalyst	NO_3^- reducing			NO_2^- reducing	
	Percentage	Mass converted (mg N/h)	N_2 selectivity	Percentage	Mass converted (mg N/h)
None	30%	0.75	30.13%	41%	1.03
RuO_x	30%	0.75	13.48%	44%	1.10
Ag	34%	0.85	19.16%	49%	1.23
Au	36%	0.90	24.68%	51%	1.28
Pd	59%	1.48	30.25%	65%	1.63
Pt	61%	1.53	35.16%	74%	1.85
Pd-Pt	96%	2.40	59.93%	100%	2.50

Table S2a Investigation of apparent quantum yields at different beam light intensities for $ZnCr_2S_4$-0.5wt% Pd-0.5 wt% Pt. Experimental conditions: 100 mL of NO_3^- aqueous solution containing 50 ppm N, 0.1 g catalyst, 500 W Hg lamp.

Beam intensity (W)	Conversion percentage	N_2 selectivity	Nitrogen converted (mg N/h)	AQY (%)
1.77	25.8%	29.3%	1.29	0.54
1.63	23.1%	27.2%	1.16	0.76
1.50	20.1%	24.9%	1.00	0.86
1.34	16.3%	21.2%	0.82	1.12
1.13	11.4%	17.3%	0.57	1.54
0.92	5.0%	11.9%	0.25	2.15

Table S2b Investigation of apparent quantum yields at different beam light intensities for $ZnCr_2S_4$-0.5wt% Pd-0.5 wt% Pt. Experimental conditions: 100 mL of NO_3^- aqueous solution containing 50 ppm N, 0.25 g catalyst, 500 W Hg lamp.

Beam intensity (W)	Conversion percentage	N_2 selectivity	Nitrogen converted (mg N/h)	AQY (%)
1.77	48.7%	60.3%	2.43	1.23
1.63	46.9%	57.9%	2.34	1.57
1.50	39.3%	51.3%	1.94	1.73
1.34	31.4%	42.0%	1.57	2.18
1.13	21.8%	33.4%	1.09	2.90
0.92	9.2%	22.4%	0.46	3.73

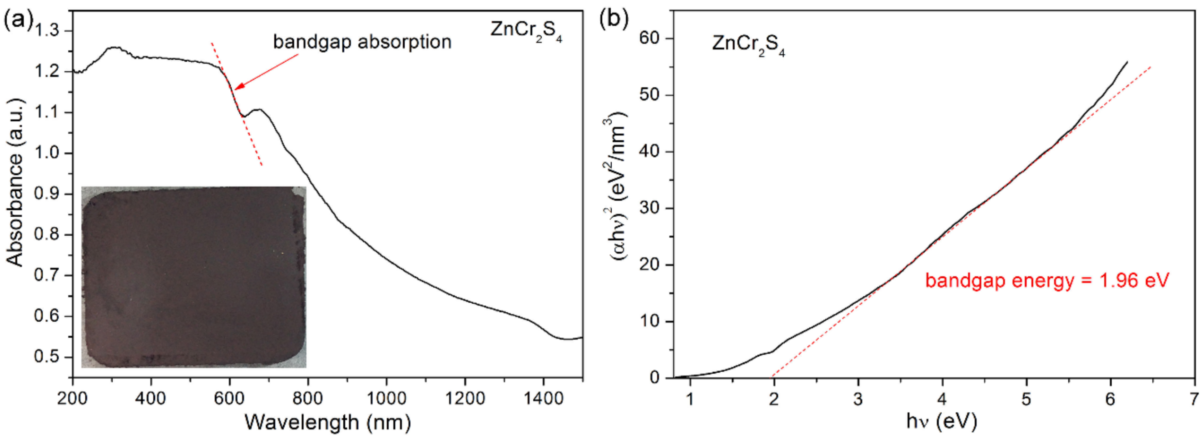

Fig. S1 (a) UV-vis diffused reflectance spectrum for as-prepared $ZnCr_2S_4$. Insert is the photograph of the powder sample. (b) The plot of $(\alpha h\nu)^2$ against hν (assuming it is a direct transition). The extrapolated value of hν at $\alpha = 0$ gives an absorption edge energy corresponding to E_g, which is 1.96 eV for $ZnCr_2S_4$.

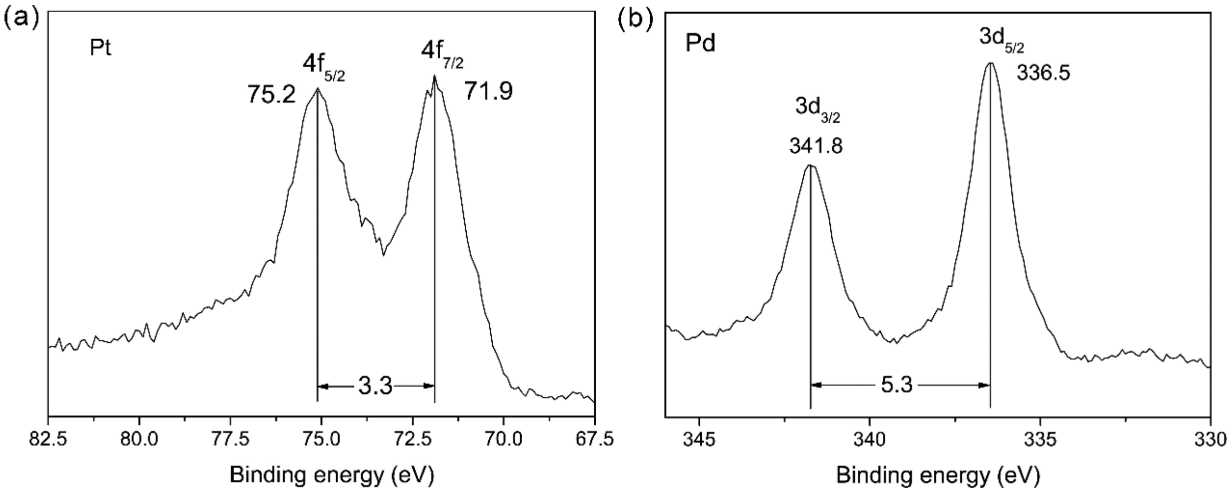

Fig. S2 XPS spectrum for $ZnCr_2S_4$-0.5 wt% Pt-0.5 wt% Pd.

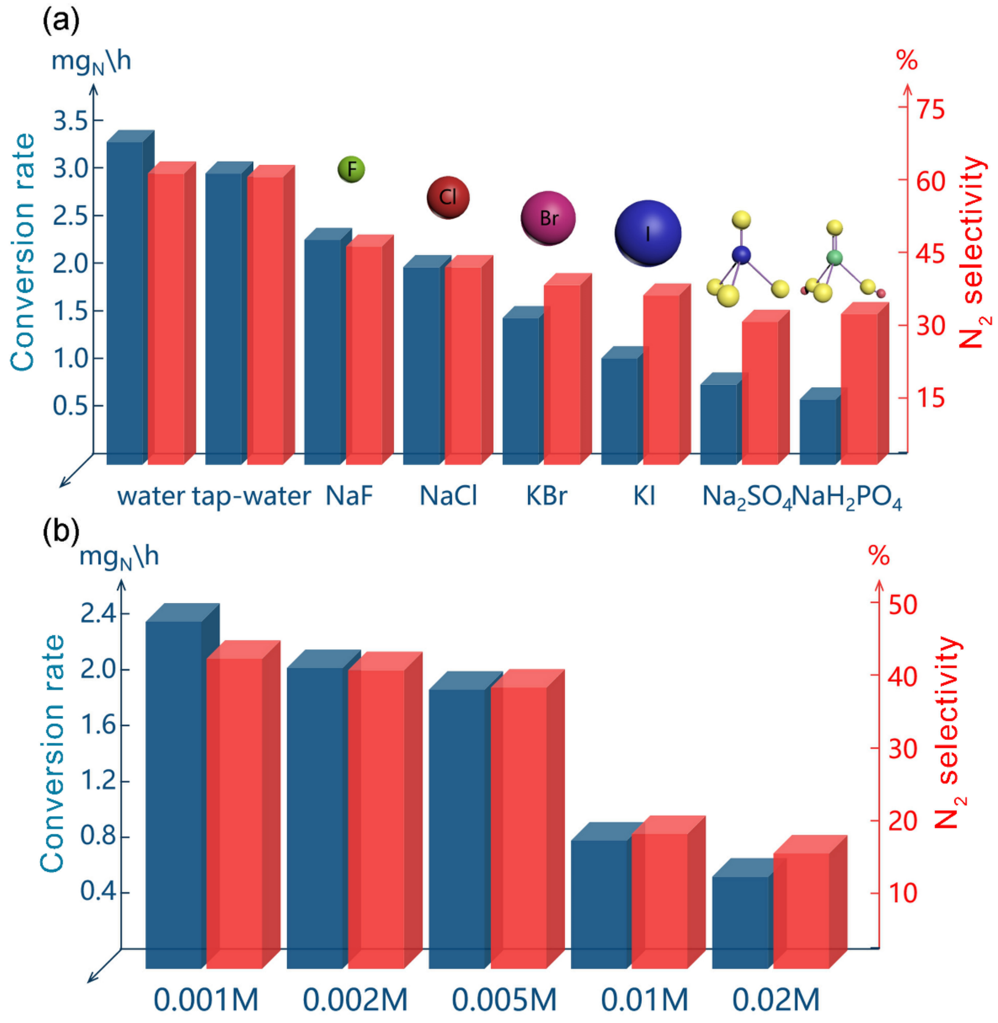

Fig. S3 (a) Photocatalytic conversion rate and N_2 selectivity in pure water, tap water, and the aqueous solutions with additional salts (0.001 M) over $ZnCr_2S_4$-1 wt% Pd-1 wt% Pt. (b) Photocatalytic conversion rate and N_2 selectivity in the aqueous solutions with NaCl in different concentrations. Note all the reactions were performed in sodium oxalate solution. Experimental conditions: 100 mL of NO_3^- aqueous solution containing 100 ppm N, 0.1 g photocatalyst of $ZnCr_2S_4$ loaded with 1 wt% Pd-1 wt% Pt, outer irradiation from 500 W Hg lamp, sodium oxalate as the sacrificial agent, in evacuated system.

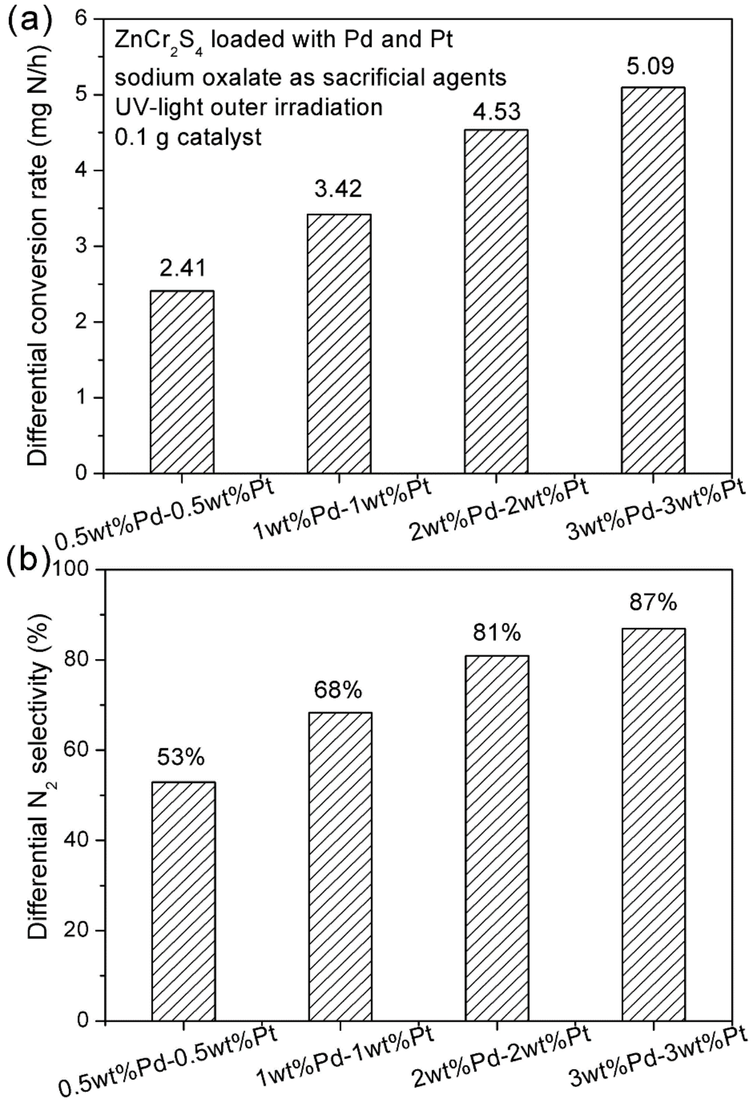

Fig. S4 (a) Differential conversion rates and (b) N_2 selectivity for $ZnCr_2S_4$ loading with both Pd and Pt. Photocatalytic conditions: 100 mL of NO_3^- aqueous solution containing 50 ppm N, 0.1 g of catalyst, 500 W Hg lamp.

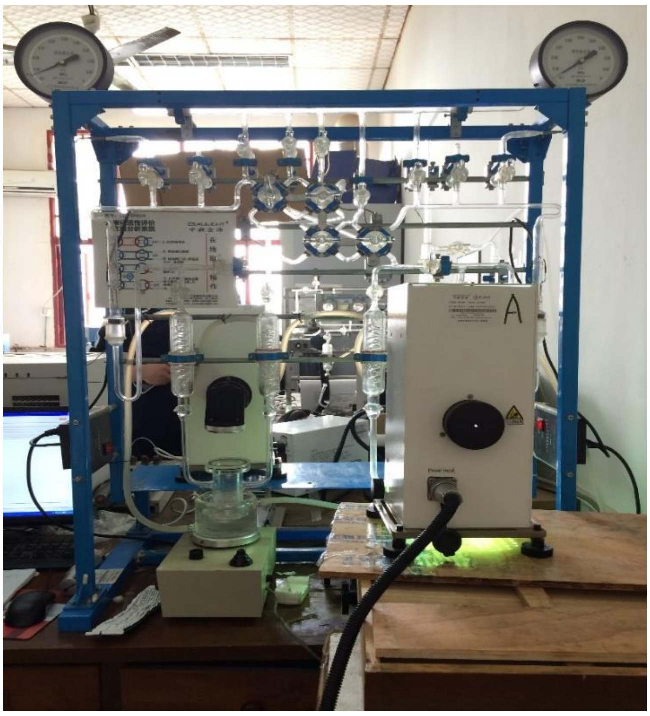

Fig. S5 Photograph of the photocatalytic evaluation setup for out irradiation in our work.

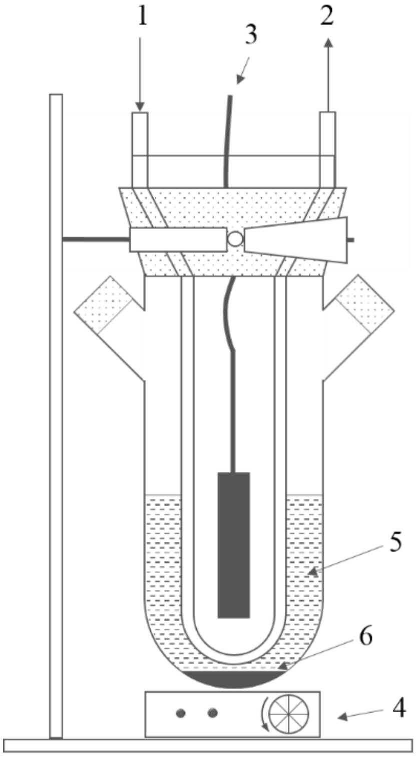

Fig. S6 Schematic view of the photocatalytic setup with an inner irradiation. 1, water in; 2, water out; 3, Xe-lamp; 4, magnetic stirrer; 5, reaction solution; 6, catalyst powder.
